# How can behavioural science help us design better trials?

**DOI:** 10.1186/s13063-021-05853-x

**Published:** 2021-12-04

**Authors:** Katie Gillies, Jamie Brehaut, Taylor Coffey, Eilidh M. Duncan, Jill J. Francis, Spencer P. Hey, Justin Presseau, Charles Weijer, Marion K. Campbell

**Affiliations:** 1 Health Services Research Unit, University of Aberdeen, Aberdeen, UK; 2 Clinical Epidemiology Program, Ottawa Hospital Research Institute (OHRI), The Ottawa Hospital, General Campus, 501 Smyth Rd, Ottawa, ON K1H 8L6 Canada; 3 School of Epidemiology and Public Health, University of Ottawa, Ottawa, ON K1G 5Z3 Canada; 4 Melbourne School of Health Sciences, University of Melbourne, Melbourne, Australia; 5 Harvard Center for Bioethics, Harvard University, Boston, USA; 6 Prism Analytic Technologies, Cambridge, MA USA; 7 Departments of Medicine, Epidemiology & Biostatistics, and Philosophy, Western University, London, Ontario Canada

## Background

Clinical trials remain the cornerstone of evidence-based health care. As of July 1, 2021, there were 382,313 clinical trials registered on ClinicalTrials.gov, an average of 33,400 new registrations over each of the past 3 years, and 19,782 new registrations for this year (2021) alone [1]. Even assuming modest sample sizes of 125 participants for each of those new 19,782 trials in that one registry, these trials already require more than 2.4 million participants this year, approximately 13,737 every day, and hundreds of potential participants being approached or being followed up right now.

Despite the incredible volume of research activity and collective trial experience, trials still routinely take longer (and cost more) than originally proposed, often due to challenges with recruitment (including participants in a trial) and/or retention (keeping participants in a trial) [2]. For example, only 56% of UK National Institute for Health Research Health Technology Assessment funded trials recruited the number of people they needed, and some suffered loss to follow-up of up to 77% [2]. Alongside challenges of recruitment and retention, many other trial process-related deficiencies produce trial results that are at best unreliable and at worst unusable leading to research waste [3].

Amongst the existing evidence on how to improve the design and conduct of trials, little attention has been given to the integral and multifactorial role of human behaviour to trial success. Indeed, all of these trials depend on behaviours: they rely on people (patients, clinicians, trial staff) performing actions (such as receiving or delivering a trial intervention, attending a clinic, returning a questionnaire, or approaching eligible participants) that they would not do otherwise. Clearly defining and specifying behaviours is a key first step in clarifying behaviours in terms of *who* needs to do *what* differently *to/for whom* and *when*. The AACTT behaviour specification framework was developed for implementation research and proposes five domains (action, actor, context, target, time) to describe and detail relevant behaviours [4]. The AACTT framework can be used to specify the behaviours of individuals and to describe team and organisational behaviour. Even considering a simple process such as developing AACTT specifications for key trial activities (e.g. returning a questionnaire) could provide considerable additional insight. There are many influences on participants, trial staff, and clinicians’ behaviours within clinical trials. These trial-related behaviours are widespread, often contextually dependent and amenable to change. Indeed, failure to recognise the behavioural influences (and change them where appropriate) could contribute to the failure of the trial. Moreover, insofar as behaviours are at the heart of clinical trial delivery, then behavioural science—the study of behaviour and behaviour change—can provide critical, replicable, and generalisable insights for the clinical trials community.

## The potential value of behavioural science to inform trial design, delivery, and reporting

Behavioural science is cross-disciplinary and has been considered as an umbrella term that includes contributions from various disciplines (including psychology, economics, sociology, political science, and anthropology). The field concerns how and why people behave as they do. Behavioural science as applied to health seeks to use the theories, methods, and knowledge from these disciplines to design more effective health care interventions. Within this article, we have focussed primarily on contributions from psychology but recognise that many of the other disciplines may have important contributions for clinical trials*.* The application of behavioural science to complex problems in health care has clearly been effective in changing both patient (e.g., smoking cessation) and health care professional behaviour (e.g., following recommendations for acute stroke care) as well as improving patient outcomes on both the short and long terms [5, 6]. For decades, implementation science has informed how to improve the uptake of trial results into practice, but lessons from the wider field of behavioural science have only recently been applied to problems of trial design and delivery.

Clinical trials are complex and made up of multiple processes at various stages of the trial lifecycle. These include, but are not limited to, question conception, trial design, grant and protocol writing, planning trial delivery, recruitment, intervention delivery, data collection, retention, analysis, dissemination of findings, and closedown. Understanding the influences on trial processes as multiple behaviours (performed by multiple actors), across the trial life cycle, has the potential for developing more effective evidence-based strategies for improvement. For example, recruitment can be further broken down to designing recruitment marketing materials (performed by investigator teams), approaching all eligible participants (performed by trial recruiters), signing of the consent form (performed by recruiters and participants), etc. Once a trial process is broken down in this way, it becomes more amenable to study and improvement with the tools of behavioural science. In what follows, we will present detailed examples of how behavioural science has been applied to trial processes in just this way.

### When is a trial needed?

It can be difficult to determine when the evidence is strong enough to support the widespread implementation of an intervention or when further RCTs may be required. A study by Cuthbertson and colleagues sought to identify why the ICU community had not widely adopted the use of selective decontamination (SDD) of the digestive tract in ICU patients given the substantial evidence supporting the effectiveness of SDD from 12 meta-analyses of 36 RCTs [7]. Using a Delphi survey based on the Theoretical Domains Framework (TDF), the team were able to assess the factors affecting the clinical behaviour and the appetite for a further RCT [7]. In brief, the TDF is a comprehensive framework that proposes 14 theoretical domains that may influence behaviour (e.g. knowledge, behavioural regulation, emotion) [8]. Priority domains can be determined with regard to facilitators or barriers to performing the behaviour, which are then targeted when developing behavioural interventions [9]. The Delphi study concluded that the behaviours (in this case actively delivering SDD to ICU patients) would not be more widely implemented without further supportive evidence given the concern regarding the lack of appropriate/relevant outcomes in the existing trial contexts. This work directly informed the successful funding of an international trial of SDD in ICU patients with hospital mortality (primary) and antibiotic usage/resistance (secondary) as outcome measures. This approach of analysing the profile of behavioural responses to determine whether further (or indeed preliminary) trials are needed could be adapted for many clinical questions as one of the first steps in designing an RCT.

### Is a trial feasible?

There are many ways that assessments of trial feasibility can be conducted. One of the benefits of assessing trial feasibility using a behavioural science approach is that it offers detailed identification of barriers and potential facilitators to performing key behaviours, which, in turn, drive the development of highly tailored, specific solutions with real potential to overcome feasibility challenges. To inform a future large-scale evaluation of a prehospital trauma intervention, ongoing work by Gillies et al. is developing a detailed behaviour specification and ‘diagnosis’ to identify the key challenges and opportunities for improving the feasibility and ultimate success of the future trial [10]. Specifically, interviewing health care professionals who are currently (or potentially would be) delivering the intervention will allow an understanding of behavioural challenges in intervention delivery and will provide evidence to help future strategies succeed for the future randomisation of participants.

Understanding the broad challenges for potential trial participants is also an important barrier to overcome when recruiting to a trial. Some studies have used surveys informed by behavioural theory, such as the health belief model (a model that assumes people’s subjective health considerations determine health-related behaviour), to investigate why patients choose to participate in trials [11, 12]. Brehaut and colleagues have taken this a step further by developing a theory-guided TDF survey to identify the challenges and opportunities to trial participation amongst potential participants, rather than amongst those who have participated [13]. The use of tailored surveys (which could be informed by the Brehaut approach) can be applied pretrial to determine what the main barriers to trial recruitment are likely to be and to facilitate recruitment strategies to address these barriers.

### Do trial teams involve patients and public partners?

There are many motivators for involving patients and/or the public as research partners, not least of all to ensure the research is relevant for those it seeks to serve. The involvement of the patient and public partners in trials is now commonplace, but the extent and depth of that involvement vary significantly. A recent study by Goulao et al. surveyed trial teams to investigate the behavioural determinants of involving patient partners in numerical aspects of trials using a TDF-based survey [14]. The survey highlighted several domains that act as barriers (knowledge; skills and beliefs about capabilities; resources; reinforcement) which could be targeted with behaviourally specified interventions to improve current practice. This approach could be extended to the involvement of other stakeholders in the trial design and delivery process.

### What are the challenges to trial recruitment?

Recruitment to clinical trials has been identified as the top methodological priority by UK Clinical Trials Units directors, evidencing its importance to many in the community [15]. Understanding the main challenges relating specifically to trial recruitment has been the focus of much research, but still very few high-quality, generalisable solutions exist [16]. A number of studies have applied behavioural science to understand the problems of trial recruitment. This has included conducting behavioural theory-informed qualitative interviews to understand the potential challenges to recruitment to early phase trials from the perspectives of clinicians and patients [17–20]. Findings from these studies were then used to refine the design and conduct of future trials. In addition to early phase trials, an exploratory TDF-based approach is currently being used to understand the challenges faced by health care professionals when recruiting pregnant women into clinical trials. The findings of the interviews will be used to develop, and subsequently test, a behaviour change intervention targeting professionals to improve the recruitment of pregnant women [21]. A similar approach has been used to develop an implementation intervention to address low recruitment to cancer clinical trials amongst rural and minority community urology practices [22]. This implementation intervention, termed ‘learn/inform/recruit’ was deemed appealing and acceptable by stakeholders [22]. The theory of planned behaviour (which proposes a model based on three variables: attitudes, subjective norms, and perceived behavioural control, which work together to predict the intention to perform a behaviour) has also been used to explore trial recruitment [23, 24]. TPB was used as a guiding framework to assess an intervention aimed at supporting patients in making fully informed decisions about lung cancer trials, highlighting that the application of this approach can be used with a range of theoretical approaches [23]. Using theoretical frameworks in this way is helpful for the individual trials as it enables more direct identification of possible strategies/techniques tailored to address the construct/factors more readily. A further advantage is this approach also allows the opportunity to combine data across studies and consider the meta-level findings of relevance across (possibly similar phased) trials.

Whilst many of the examples to date have been based on the TDF, a range of other behavioural theories and frameworks have been applied to problems of trial recruitment and retention. A recent mapping review identified 31 studies that used a range of theories/frameworks including the TDF, the theory of planned behaviour, social cognitive theory (describes the influence of the actions of others, experiences, and environmental contexts on an individual’s health behaviour), and others [Coffey et al. manuscript under review [25, 26]]. Establishing whether there are ‘best fit’ theories and frameworks for different trial problems is an important consideration for future work in this area.

### How is the trial intervention delivered?

Process evaluations have long been embedded in randomised evaluations of clinical interventions to understand various aspects of delivery [27]. Many of these have included behavioural theories that have underpinned the behaviour change interventions being evaluated or indeed used theories (from a wide range of fields) to understand the mechanisms of change or barriers to implementation. However, less well addressed in this literature is the application of behavioural science to unpack the behaviours and behaviour change required for the delivery of clinical interventions within trials. Two recent studies have aimed to do just that. The first was with health care professionals delivering a trial of individualised temperature-reduced haemodialysis to explore the behaviours involved in adjusting the temperature on a dialysis machine [28]. The second was using a theory-based approach in data analysis gathered from both health care professionals and patients to explore trial experience and beliefs and experiences of the intervention, which in this case is catheter wash out policies [29].

### What are the challenges to trial retention?

Similar to work on recruitment, a number of studies are now emerging that have conducted qualitative interviews informed by behavioural frameworks to understand trial retention behaviours such as postal questionnaire return and follow-up clinic attendance [30, 31]. Findings from the interview studies were then used to develop participant-centred, theory-informed interventions to promote trial retention that have been codesigned with stakeholders and will be tested in randomised evaluations [32].

The Cochrane reviews on interventions to improve recruitment to and retention in clinical trials have found very little evidence of effect [16, 33]. The reviews largely include interventions that were not designed as behaviour change interventions (BCIs) with only a minority (< 5%) conceptualised as BCIs, yet the implicit aim of the majority is to change participants’ recruitment or retention behaviour. For example, intervention categories in both reviews include incentives and rewards (which target the theoretical behavioural domain of reinforcement), reminders and prompts (target theoretical domain of memory, attention and decision-making, environment context, and resources), and improvements to information (target theoretical domain, knowledge). Yet, the design and delivery of these interventions do not include the explicit inclusion of behaviour change input, nor are these interventions informed by the bodies of knowledge in the behavioural sciences. Deconstructing interventions into their behaviour change techniques (BCT, defined as the smallest ‘active ingredient’ of an intervention that can be used alone or in combination) has the potential to identify possible ‘active ingredients’ which could be enhanced in future replications of evaluations or implementation [34]. Duncan et al. demonstrated the potential value of this approach with preliminary work identifying BCTs within interventions shown to improve retention [35]. The findings identified that BCTs were used amongst the interventions but not labelled as such (notably incentives and prompts—both behavioural strategies) and that several implicit BCTs were applied in both intervention and control strategies. The need to explicitly incorporate BCTs during the design of interventions to target recruitment and retention behaviours (and others relevant for trial conduct) is key. A small number of studies have developed behaviour change interventions for trial retention by incorporating BCTs into covering letters of questionnaires, newsletters, and also use of trial stickers on envelopes (to act as prompts) [36]. Preliminary evaluations of these behaviourally focussed trial process interventions are showing promise, but replication and further research to include patient input and assessment are required to maximise their potential [33, 36]. Creating a shift in the conceptualisation of recruitment and retention interventions to be considered (during design and delivery) as behaviour change interventions may provide more potential for more focused assessment of effectiveness and may enhance replicability.

**Fig. 1:**
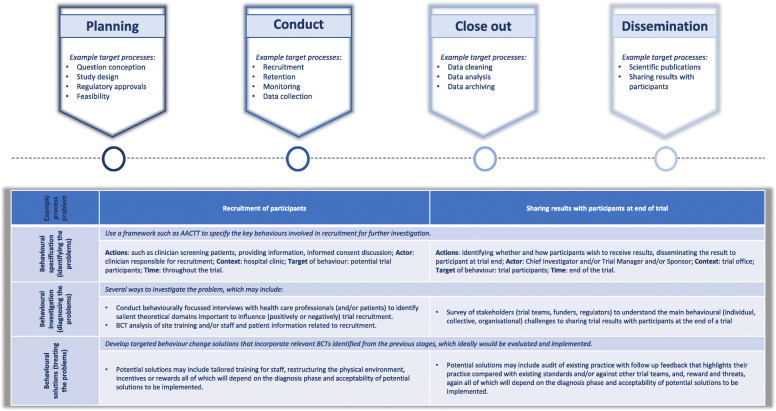
Trial lifecycle highlighting example trial processes and potential application of the behavioural science approach

It is important to highlight that the examples provided here are not an exhaustive list but are exemplars from key trial life cycle stages that serve to show where existing empirical studies have demonstrated the potential for a behavioural approach to address trial process problems (see Fig. 1). In particular, the challenges that many trials have faced during the COVID-19 pandemic (such as the move to remote delivery of recruitment, interventions, and follow-up) also provide a wealth of opportunities to apply behavioural approaches to generate evidence-informed solutions from the perspective of trial teams, regulators, and trial participants. A varied range of other trial process problems could also benefit from this approach including (but not limited to) choosing outcomes, participants’ experience, and sharing of trial results with trial participants. In addition, several behavioural approaches such as multiphase optimisation strategies (MOST), intervention mapping, and ‘nudging’ (a recent focus in the behavioural economics literature) could also warrant investigation in the future [37–39]*.*

## Core considerations for applying behavioural approaches to trials

It is of greater importance than ever to ensure that any strategies or approaches used in trials are sensitive to the different needs of diverse trial populations. For example, the majority of interventions targeting trial recruitment and retention to date have been developed by and tested in largely White populations [16, 33]. A recent mapping review of the published studies that used behavioural strategies to understand or develop solutions to problems of trial recruitment and/or retention identified that 35% of studies (*n* = 11) were set within underserved populations (Coffey et al., manuscript submitted [25]). This may suggest the potential for behavioural approaches to begin to address some aspects of inclusion of underserved communities in trials, ensuring that future research considers equitable participation for all [40]. However, the systemic structural and institutional challenges of ensuring opportunities and access to research are available for all and will also require work that may extend beyond a behavioural framework.

Similarly, much of the work in this space is being conducted in developed countries, but there are now projects being developed which also plan to use a behavioural science approach in trials in low- and middle-income countries. One such study is focussing on the behaviours of postal questionnaire return and follow-up clinic attendance after surgery in a number of LMICs (e.g. India, South Africa, Philippines). This project will apply the capabilities, opportunity, and motivation behaviour system (COM-B) to provide a behavioural diagnosis and identify intervention functions that then help to assess the relevance of existing interventions to modify target behaviours and as such ‘treat’ the problems [41].

It is also worth considering not just how behavioural science can maximise learning opportunities at key stages of trial design and delivery, but also its potential value across different phases of trials and trials of various intervention types, e.g. clinical trials of investigational medicinal products (CTIMPs) and non-CTIMPS. For example, we know that the motivations of participants to participate first in human studies are largely different to the motivations of those who participate in later stage pragmatic effectiveness trials and are often linked to risk [42, 43]. It may also be that these approaches could be more or less acceptable for trials in particular clinical contexts (e.g. emergency care) or populations (e.g. children or adults who lack capacity). Considerations of risk, whether in relation to the stage of evaluation or the interventions under investigation, also raise another important consideration.

There will of course be some core challenges for trial teams in applying a behavioural approach to trials. Some of these may relate to trial teams lacking confidence or knowledge in how to apply particular theories or frameworks. The best option would likely be to include behavioural scientists as part of the trial team but failing that, tools that make this approach accessible and implementable will be key. A good starting point exists amongst key papers for health behaviour change, in particular, some worked examples of how to apply the AACTT framework [4], a step-by-step guide to using the TDF [9], a guide for constructing questionnaire informed by the TPB [44], and a core textbook on the behaviour change wheel which covers COM-B interventions and includes lots of practical examples of application albeit in a different context [45].

The ethics of behaviour change interventions requires further exploration. Will certain behavioural approaches, and therefore particular behaviour changes interventions, be more ethical in some trials over others? For instance, the use of ‘nudges’ in informed consent has been criticised on ethical grounds [46]. But should behaviour change interventions be conceptualised as nudges, and outside of the consent process, what is their impact on the autonomy of trial participants? Where are the limits to when behaviour change interventions become more or less acceptable, practically and philosophically, in the context of trial-related behaviours?

One ethical advantage to framing trial processes as complex behaviours is that, by unpacking the various behaviours, actors, and influences involved in any process, it becomes clear how trial success is a social phenomenon—that is, trial funders, investigators, recruiters, and patients all need to act in particular ways at particular times in order for the trial to achieve its scientific and social aims. For example, the model of the recruitment process that we described above includes behaviours, and therefore potential interventions, that would target investigators, recruiters, or patients. By contrast, the existing literature on nudges in trials has tended to focus almost exclusively on interventions that would target patient behaviour—who are often going to be the most vulnerable stakeholders. Whilst focusing on patient behaviour is certainly important, by widening the behavioural lens, so to speak, the model we advocate opens up possibilities to study and improve trial behavioural with a more holistic, and potentially more equitable, approach.

Lastly, it will be important to ensure that behavioural interventions or approaches seeking to address trial problems are evaluated using robust approaches such as Studies Within A Trial (SWATs), or other appropriate study designs, to determine the effectiveness and mechanisms of effect and to identify potential behavioural confounders in trial conduct [47]. Ensuring interventions and processes for evaluation are detailed and pre-specified in publicly available protocols will help to encourage replication by other teams across a range of trials.

## The added value of behavioural approaches to trial design and conduct

There is considerable potential for the behavioural approach to trials in that it offers significant flexibility. For example, this approach can be applied before a trial is started, can be implemented with multiple methods, and is theory-informed. A further added value component of developing the methodology around behavioural optimisation and operational strategies for clinical trials will be the greater potential for sharing resources and learning across data sets. The potential for aggregation of diagnostic behavioural data across trial types, collected using the same tools, to explore the over-arching challenges and opportunities facing clinical trials has huge potential. Further enhancing the potential for learning across trials and the application of transferable solutions should be a focus going forward. Current research to determine the best practices for sharing qualitative data in clinical trials will demonstrably help move this agenda forwards [48].

## Conclusions

Trialists are implicitly using behavioural approaches already. Fully engaging with the science may help to make more explicit decisions for what behavioural strategies to include and why at each stage of a trial. This explicit operationalisation of a behavioural approach would enable the greater potential for generalisability and shared accumulation of evidence to ensure that the funding, and the time and effort trial teams and trial participants contribute are maximised. Ultimately, accelerating the availability of new therapies to better the health of the population. Adopting a behavioural approach to address problems of trial design and conduct has been shown to be an effective, reproducible, transparent, and generalisable approach. As this is an emerging field, however, thoughtful consideration and empirical work to establish the most suitable approaches to a range of trial problems and contexts, whilst developing implementable methodology supported by appropriate resources, is a key next step.

## Data Availability

Not applicable
